# Experiencia de 5 años en soporte circulatorio mecánico de corta duración en pacientes posinfarto de miocardio: un reporte del registro INCORMACS

**DOI:** 10.47487/apcyccv.v1i3.76

**Published:** 2020-09-30

**Authors:** Manuel Alejandro Pitta Acevedo, Christian Soplopuco Palacios, José Luis Añorga Ocmin, Miguel Ángel Lescano Alva, Ana María Sihuayro Ancco, Franz Ronald Soplopuco Palacios, José Luis Tapia Leonardo

**Affiliations:** 1 Servicio de Cardiología Clínica, Instituto Nacional Cardiovascular INCOR - EsSalud. Lima, Perú. Servicio de Cardiología Clínica Instituto Nacional Cardiovascular INCOR - EsSalud Lima Perú; 2 Servicio de Cardiología perioperatoria. Instituto Nacional Cardiovascular INCOR - EsSalud. Lima, Perú. Servicio de Cardiología perioperatoria Instituto Nacional Cardiovascular INCOR - EsSalud Lima Perú; 3 Instituto Nacional Cardiovascular INCOR - EsSalud. Lima, Perú. Instituto Nacional Cardiovascular INCOR - EsSalud Lima Perú; 4 Servicio de Cirugía Cardiaca. Instituto Nacional Cardiovascular INCOR - EsSalud. Lima, Perú. Servicio de Cirugía Cardiaca Instituto Nacional Cardiovascular INCOR - EsSalud Lima Perú

**Keywords:** Infarto del Miocardio, Choque Cardiogénico, Oxigenación por Membrana Extracorpórea, Myocardial Infarction, Shock, Cardiogenic, Extracorporeal Membrane Oxygenation

## Abstract

**Objetivo.:**

Determinar las características clínicas y epidemiológicas de los pacientes con dispositivos de soporte mecánico circulatorio (DSMC) de corta duración, posinfarto de miocardio (IAM) en un hospital de referencia nacional.

**Materiales y métodos.:**

Estudio descriptivo, retrospectivo, de pacientes pos-IAM en los que se implantó dispositivos de soporte mecánico circulatorio de corta duración desde el año 2015 al 2020.

**Resultados.:**

Se implantaron nueve dispositivos de asistencia en igual número de pacientes debido a choque cardiogénico pos-IAM. Todos los pacientes fueron varones con IAM con elevación del segmento ST (IAMCEST). Se implantaron ocho dispositivos de oxigenación por membrana extracorpórea (ECMO) y una bomba de flujo centrífugo (en configuración LVAD). La mediana de tiempo de uso del dispositivo fue de ocho días. Las complicaciones más frecuentes fueron la hemorragia no fatal (55,6%), injuria renal aguda (44,4%) y la sepsis (44,4%). La mortalidad intrahospitalaria fue de 55,6%.

**Conclusiones.:**

El uso de DSMC de corta duración posinfarto de miocardio es aún limitado en nuestra institución y tiene como principal representante al ECMO pos-IAMCEST complicado con choque cardiogénico. A pesar del uso de estos dispositivos la mortalidad intrahospitalaria es elevada.

## Abstract (en)

**Objective.:**

To determine the clinical and epidemiological characteristics of patients with short-term mechanical circulatory support devices (MCSD) after myocardial infarction (MI) at a national referral hospital.

**Materials and methods.:**

Descriptive, retrospective study of post-MI patients in whom short-term mechanical circulatory support devices were implanted from 2015 to 2020.

**Results.:**

Nine mechanical support devices were implanted, in the same number of patients due to cardiogenic shock post MI. All patients were men with ST-elevation myocardial infarction (STEMI). Eight extracorporeal membrane oxygenation devices (ECMO) and one centrifugal flow pump (in LVAD configuration) were implanted. The median time of use of the devices was eight days. The most frequent complications were non-fatal bleeding (55.6%), acute kidney injury (44.4%) and sepsis (44.4%).In-hospital mortality was 55.6 %.

**Conclusions.:**

The use of short term MCSD after myocardial infarction is still limited in our institution and its main representative is the ECMO post STEMI complicated by cardiogenic shock. Despite the use of these devices, in-hospital mortality is high.

Los pacientes con infarto agudo de miocardio (IAM) pueden desarrollar múltiples complicaciones, entre ellas insuficiencia cardiaca aguda que puede llegar hasta el choque cardiogénico (CC), este se define como la disfunción contráctil miocárdica, asociada a un gasto cardiaco inefectivo que ocasiona colapso circulatorio e hipoperfusión tisular persistente ^1^^,^^2^. En estas circunstancias, el manejo conservador del CC con inotrópicos y vasopresores está asociado a serias limitaciones, incluyendo arritmias, aumento del consumo de oxígeno miocárdico, siendo en muchos casos insuficiente, lo cual conlleva a una mortalidad del 40 - 50%, a pesar de los avances en la terapia médica e intervención coronaria percutánea ^2^^-^^4^.

Actualmente el desarrollo y avances en los dispositivos de soporte mecánico circulatorio (DSMC) hacen posible ofrecer una alternativa en el manejo de esta entidad. Diversos estudios observacionales y aleatorizados han evaluado los resultados de estos dispositivos en pacientes posinfarto, con resultados muy variados, y aún no está claro cuál es el soporte ideal del IAM complicado con falla cardíaca, CC y/o ruptura de septo interventricular ^5^^,^^6^.

Dado que los DSMC constituyen un avance importante en el manejo de pacientes con complicaciones posinfarto de miocardio, su uso ha aumentado en los últimos años en Perú y el mundo. Ante la ausencia de datos publicados en nuestro país, este reporte tiene como objetivo determinar las características clínicas y epidemiológicas de pacientes a quienes se implantó DSMC por IAM, así como las complicaciones y evolución intrahospitalaria en un hospital de referencia nacional.

## Materiales y métodos

Se realizó un estudio descriptivo, retrospectivo, en el cual se incluyeron de manera consecutiva pacientes mayores de 18 años a quienes se implantó DSMC de corta duración después de un IAM, desde enero del año 2015 a marzo del 2020; los datos se obtuvieron del registro nacional INCORMACS (registro de soporte mecánico circulatorio de corta duración en el Instituto Nacional Cardiovascular - INCOR, Lima-Perú). Se incluyeron datos epidemiológicos del paciente (edad, sexo, peso), diagnóstico e indicación del dispositivo, así como información de resultados del paciente (éxito del soporte, complicaciones y mortalidad intrahospitalaria).

 Se definió sangrado como la presencia de uno o más de hemorragia gastrointestinal, sangrado en la canulación y/o en sitio quirúrgico; complicaciones neurológicas como: accidente cerebrovascular isquémico/hemorrágico; complicaciones renales: aumento de creatinina > 1,5 mg/dL y/o terapia de reemplazo renal; complicaciones infecciosas: sepsis, infección documentada o sospechada más parámetros de disfunción orgánica y complicaciones periféricas como trombosis de miembros. El retiro exitoso del dispositivo se definió por la ausencia de inestabilidad hemodinámica y choque cardiogénico, que permitió el cese del soporte o el trasplante cardiaco.

Las variables cualitativas se expresaron en frecuencias y porcentajes, mientras que las cuantitativas en mediana y rango intercuartil (RIQ) al ser variables que no siguen una distribución normal. Para procesar la información se utilizó el programa estadístico Microsoft Excel versión 16.33 - 2019. El trabajo cumplió integramente las normas establecidas por el Comité de Ética e Investigación de nuestra institución.

## Resultados

Durante el periodo de estudio se colocaron en el INCOR 43 dispositivos de soporte mecánico circulatorio en 39 pacientes; de ellos, nueve dispositivos fueron implantados (en el mismo número de pacientes) debido a CC por infarto de miocardio con elevación del segmento ST (IAMCEST) lo que representa el 20,9% del total, siendo esta nuestra población de estudio.

Las caracteristicas clínicas básales, tipo de infarto, así como el uso de balón de contrapulsación intraaórtico (BCIAo) y ventilación mecánica (VM) se presentan en la **(**Tabla 1**)**. Se encontraron tres casos de defecto septal interventricular pos-IAM, en los cuales se colocó dispositivo de oxigenación por membrana extracorpórea (ECMO) como puente a cirugía de reparación del defecto.

**Tabla 1:** Características basales de la población de estudio

	ECMO (n = 8)	Bomba centrífuga (n = 1)	Total (n = 9)
Edad (años)*	58 (48-71)	57	58 (48-71)
Masculino, n (%)	8 (88,9)	1	9 (100)
HTA, n (%)	5 (62,5)	1	6 (66,7)
DM, n (%)	5 (62,5)	-	5 (55,6)
Tabaquismo, n (%)	4 (50)	-	4 (44,4)
Dislipidemia, n (%)	4 (50)	1	5 (55,6)
Obesidad, n (%)	3 (37,5)	-	3 (33,3)
IMACEST, n (%) -Cara anterior, n (%) -Cara Inferior, n (%)	8 (100) 4 (50) 4 (50)	1 1 -	9 (100) 5 (55,6) 4 (44,6)
BCIAo previo, n (%)	8 (100)	-	8 (88,9)
VM al implante, n (%)	6 (75)	-	6 (66,7)
PCR previa, n (%)	1 (12,5)	-	1 (11,1)

*Mediana (rango intercuartil)

HTA: hipertensión arterial. DM: diabetes mellitus. IMACEST: infarto de miocardio con elevación del segmento ST. BCIAO: balón de contrapulsación intraaórtica. VM: ventilación mecánica. PCR: parada cardiorrespiratoria.

El objetivo del implante del dispositivo fue como puente a recuperación en el 88,9% de los casos (ocho dispositivos ECMO) y en un caso como puente a trasplante (bomba centrífuga en configuración de LVAD). La mediana de tiempo de duración del dispositivo fue de ocho días. El retiro exitoso del soporte se logró en siete pacientes (77%), seis ECMO y un LVAD. Las características de los dispositivos implantados, así como los desenlaces, se presentan en las Tablas 2**y**3, respectivamente.

**Tabla 2:** Características del implante del dispositivo

Paciente	Indicación	Objetivo	Dispositivo	Tipo de dispositivo	Duración (días)	Acceso	Escala INTERMACs
1	Choque cardiogénico	Recuperación	ECMO	Cardiohelp	8	Mixta	1
2	Choque cardiogénico: infarto de VD	Recuperación	ECMO	Cardiohelp	16	Central	1
3	Choque cardiogénico: infarto de VD	Recuperación	ECMO	Cardiohelp	9	Periférico	1
4	Choque cardiogénico	Recuperación	ECMO	Cardiohelp	6	Periférico	1
5	Choque cardiogénico + CIV	Recuperación	ECMO	Cardiohelp	7	Periférico	1
6	Falla cardiaca posinfarto	Trasplante	BC	Levitronix Centrimag	1	LVAD	3
7	Choque cardiogénico + CIV	Recuperación	ECMO	Cardiohelp	7	Periférico	1
8	Choque cardiogénico + CIV	Recuperación	ECMO	Cardiohelp	10	Periférico	1
9	Choque cardiogénico	Recuperación	ECMO	Cardiohelp	8	Periférico	1

VD: ventrículo derecho. BC: bomba centrífuga. CIV: comunicación interventricular. ECMO: dispositivo de oxigenación por membrana extracorpórea.

**Tabla 3:** Desenlaces del implante del dispositivo

Paciente	Evento final	Retiro exitoso del soporte	Muerte intrahospitalaria	Causa de muerte
1	Muerte	No	Sí	Choque séptico
2	Muerte	No	Sí	Hemorragia intraalveolar
3	Recuperación	Sí	No	No aplica
4	Recuperación	Sí	Sí	Choque séptico
5	Recuperación	Sí	No	No aplica
6	Trasplante	Sí	No	No aplica
7	Recuperación	Sí	No	No aplica
8	Recuperación	Sí	Sí	Choque séptico
9	Recuperación	Sí	Sí	Choque séptico

 Las complicaciones más frecuentes durante el uso del dispositivo fueron: sangrado en cinco pacientes, e injuria renal aguda que requirió hemodiálisis y sepsis en cuatro pacientes. La mortalidad intrahospitalaria fue de 55,6% (cinco pacientes), siendo la causa mas frecuente el choque séptico **(**Tabla 4). En el grupo de pacientes ususarios de DSMC por defecto septal interventricular pos-IAM, la mortalidad fue de 33%.

**Tabla 4:** Complicaciones y mortalidad intrahospitalaria

	ECMO (n = 8)	Bomba centrífuga (n = 1)	Total (n = 9)
Sangrado, n (%)	4 (50)	1	5 (55,6)
Accidente cerebrovascular, n (%)	2 (25)	-	2 (22,2)
Trombosis de MMII, n (%)	1 (12.5)	-	1 (11,1)
Injuria renal aguda, n (%)	4 (50)	-	4 (44,4)
Sepsis, n (%)	4 (50)	-	4 (44,4)
Mortalidad intrahospitalaria n (%)	5 (62,5)	-	5 (55,6)

MMII: miembros inferiores. ECMO: dispositivo de oxigenación por membrana extracorpórea.

## Discusión

La bomba de flujo centrífuga y el dispositivo de oxigenación por membrana extracorpórea (ECMO) son las opciones de soporte circulatorio mecánico disponibles en la salud pública del Perú. El ECMO proporciona un apoyo cardiopulmonar completo, que extrae sangre venosa y la devuelve al sistema arterial, lo que apoya la función del corazón defectuoso, mientras que también oxigena la sangre mediante un oxigenador de membrana. Puede insertarse de forma periférica o central, pero requiere niveles estrictos de anticoagulación y, potencialmente, puede proporcionar una descarga ventricular izquierda incompleta; mientras que la bomba de flujo centrífuga proporciona un soporte ventricular completo, permite una descompresión y movilización uni o biventricular eficaz, pero se necesita un abordaje quirúrgico ^5^^-^^8^.

Este es el primer estudio a nivel nacional que aborda el uso de dispositivos de soporte mecánico circulatorio posterior a un infarto de miocardio, permitiendo así documentar nuestra experiencia en el uso de esta alternativa de manejo en este escenario clínico.

La causa más frecuente de CC es el IAM, que aún tiene alta mortalidad a pesar de los avances terapéuticos ^9^^,^^10^. En este documento informamos nuestra experiencia de cinco años colocando dispositivos de soporte circulatorio, de los cuales solo el 20,9% fue posinfarto de miocardio, cifra menor a la reportada en un registro de nueve años en Estados Unidos, donde llegó al 55% ^11^, esto posiblemente se debe a que el uso de DSCM nace inicialmente en nuestra institución como apoyo a pacientes con insuficiencia cardiaca avanzada (pre y postrasplante) y poscardiotomía y, recientemente, desde fines del año 2018, se fue desarrollando la estrategia en pacientes posinfarto de miocardio.

La mayoría de nuestros pacientes tenía una edad mayor a 50 años al momento del implante del dispositivo, tal como lo reportan Vallabhajosyula *et al.* ^(^^12^ y Shah *et al*. ^11^ siendo más frecuente el sexo masculino, como se reporta en dichos trabajos. A diferencia de estudios internaciones, donde se reporta el uso de DSCM por infarto de miocardio sin elevación del segmento ST (IAMSEST) en hasta 32,7% ^12^^,^^13^, en nuestro estudio solo se encontraron pacientes con diagnóstico de IAMCEST, esto podría explicarse porque el INCOR es de referencia nacional y local de IAMCEST, predominantemente.

Para la selección del tipo de dispositivo por usar en pacientes con injuria cardiaca aguda y profunda, potencialmente reversible, como en el IAM y la miocarditis, el ECMO venoarterial puede proporcionar un soporte como puente a recuperación; en aquellos con un infarto de miocardio masivo, puede ser usado como puente a una terapia de destino, tal como un dispositivo de soporte mecánico circulatorio de larga duración y/o trasplante cardiaco ^14^.

 Con relación al único paciente en asistencia circulatoria con bomba centrifuga como puente a trasplante, existe poca publicación en realidades similares a la nuestra. En un reciente estudio realizado en Argentina, Giordanino *et al*.^15^ reportan la colocación de 37 dispositivos entre ECMO (14) y bomba centrifuga (23), como puente a trasplante cardiaco, 16,2% fueron colocados por infarto de miocardio.

A diferencia de la mortalidad hallada en pacientes con complicación mecánica pos-IAM, Matteucci *et al*.^16^ reportan mayor mortalidad intrahospitalaria (64,7%), debido a que en ese estudio se incluyó a pacientes con ruptura de pared libre y ruptura de músculo papilar, problemas que no se presentaron en nuestra población. Estas complicaciones tienen una mortalidad considerable, en probable relación a la corrección quirúrgica previa al soporte con ECMO.

A pesar de que existen resultados desfavorables sobre el beneficio de ciertos tipos de soporte circulatorio en CC pos-IAM (balón de contrapulsación intraaórtico e Impella) ^5^^,^^8^^,^^17^; las guías de manejo de infarto de miocardio recomiendan considerar los DSMC de corta duracion como rescate para estabilizar los pacientes, preservando la perfusión orgánica como puente a recuperación de la función miocardica, trasplante cardiaco o, incluso, terapia de destino con LVAD ^18^. En ese sentido, el uso del balón de contrapulsación intraaórtico (BCIAo), previo a la instalación del soporte circulatorio, se dio en el 88% de los casos en el contexto de choque refractario o complicación mecanica (defecto septal interventricular) tal como se recomienda en dichas guías ^18^; dato que es mucho mayor al registro realizado en Estados Unidos, donde el uso concomitante de BCIAo fue solo del 30% ^12^, esto se debe a que en nuestra realidad este dispositivo de soporte es de más fácil acceso y colocación.

 Con relación al éxito del soporte en nuestra serie, se logró retirar el dispositivo pues se consiguió el objetivo en el 77% de los casos, pero aun así, la mortalidad intrahospitalaria fue mayor debido a complicaciones principalmente infecciosas. En informes recientes del ELSO ^19^ se reporta un 51% de decanulación por recuperación del órgano, cifra diferente a nuestra serie; se debe considerar que en este análisis del registro del ELSO se incluyeron a pacientes que tuvieron arresto cardiaco previo al implante del ECMO venoarterial.

En los pacientes que recibieron soporte con ECMO la sobrevida fue de 37,5%, dato similar a los análisis del Registro ELSO, que mencionan una sobrevida del 41,4% al alta hospitalaria ^20^, porcentajes similares han sido reportados en otros estudios ^12^^,^^16^. Se reporta que la colocación del ECMO venoarterial antes de la revascularización, está asociada con menor riesgo de muerte, colocación de LVAD o trasplante cardiaco comparado a los que se les puso después ^21^, conducta que no se llevó a cabo en la mayoría de pacientes, y que puede haber influido en los resultados.

Entre las limitaciones de este estudio debemos considerar que el diseño retrospectivo impidió investigar algunas variables clínicas, hemodinámicas y laboratoriales importantes; asimismo, se realizó en un único centro, con un pequeño número de pacientes debido a los pocos años que la institución tiene esta terapia de soporte circulatorio. La fortaleza de esta investigación es aportar los primeros resultados de una terapia con una gran perspectiva en el escenario clínico del infarto de miocardio.

## Conclusión

El uso de DSMC de corta duración posinfarto de miocardio es aún limitado en nuestra institución y tiene como principal representante al ECMO pos-IMCEST complicado con choque cardiogénico. A pesar del uso de estos dispositivos la mortalidad intrahospitalaria es aún elevada.
